# Salt intakes in sub-Saharan Africa: a systematic review and meta-regression

**DOI:** 10.1186/s12963-015-0068-7

**Published:** 2016-01-11

**Authors:** Oyinlola Oyebode, Samuel Oti, Yen-Fu Chen, Richard J. Lilford

**Affiliations:** 1 Warwick Medical School, University of Warwick, Gibbet Hill Campus, Coventry, CV4 7AL UK; 2 African Population and Health Research Centre, Manga Close, Off Kirawa Road, P.O. Box 10787-00100, Nairobi, Kenya; 3 Department of Global Health, Academic Medical Center, University of Amsterdam, and Amsterdam Institute for Global Health and Development, PO Box 22700, 1100 DE Amsterdam, The Netherlands

**Keywords:** Sodium chloride, Dietary, Sodium, Africa, Systematic review

## Abstract

**Background:**

High sodium intake increases the risk of hypertension and cardiovascular diseases. For this reason the World Health Organization recommends a maximum intake of 2 g per day and a 30 % reduction in population sodium intake by 2025. However, in global reviews, data on sodium intake in sub-Saharan Africa have been limited.

**Methods:**

A systematic review was conducted to identify studies reporting sodium intake in sub-Saharan African populations. Meta-regression analyses were used to test the effect of year of data collection and method of data collection (urinary/dietary), as well as any association between sex, urban/rural status or a country’s economic development, and population sodium intake.

**Results:**

We identified 42 papers reporting 67 estimates of adult population sodium intakes and 12 estimates of child population sodium intakes since 1967. Of the 67 adult populations, 54 (81 %) consumed more than 2 g sodium/day, as did four of the 12 (33 %) child populations. Sixty-five adult estimates were included in the meta-regression, which found that urban populations consumed higher amounts of salt than rural populations and that urine collection gave lower estimates of sodium intake than dietary data.

**Conclusions:**

Sodium intake in much of sub-Saharan Africa is above the World Health Organization’s recommended maximum intake and may be set to increase as the continent undergoes considerable urbanization. Few identified studies used stringent measurement criteria or representative population samples. High quality studies will be required to identify where and with whom to intervene, in order to meet the World Health Organization’s target of a 30 % reduction in population sodium intake and to demonstrate progress towards this target.

## Background

Non-communicable diseases (NCDs) are the leading cause of global disease burden [1, 2], with 80 % of NCD mortality occurring in low- and middle-income countries (LMICs) [3]. Of these, the cardiovascular diseases (CVD), ischemic heart disease and stroke, are the leading causes of death and disability, and are increasing in prevalence [1, 2]. Whereas ischemic heart disease predominates in high-income countries, stroke is the most important CVD in African countries [4]. In 2005, 87 % of stroke death occurred in LMICs, rising to 94 % of stroke deaths in people under 70 years old [5].

Excess sodium intake raises blood pressure, leading to hypertension, the principal preventable risk factor for stroke [6, 7]. Excess sodium is also a major risk factor for other CVDs and for stomach cancer [6–8]. It is possible that populations in sub-Saharan Africa are more vulnerable to the effects of a high sodium diet than other populations due to the greater prevalence of inter-uterine growth restriction, as well as genetic factors [9, 10].

The normal (physiological) requirement for sodium is likely to be between 0.1 and 1.0 g (2.5 g salt) daily [11]. Recommended intake is less than 2.0 g sodium (5.0 g salt) for adults aged 16 and over, and this recommended maximum level of intake should be adjusted downwards in children ages 2-15 based on the energy requirements of children relative to those of adults [12]. The World Health Organization (WHO) *Global Action Plan for the Prevention and Control of Non-Communicable Diseases 2013–2020* identifies nine key targets for the reduction of chronic disease, including “a 30 % relative reduction in mean population intake of salt/sodium” [13]. The reason for a focus on a relative reduction rather than aiming to reduce salt intake to recommended levels reflects the observation that most populations have a mean sodium intake that considerably exceeds the 2.0 g recommendation [14, 15].

Given the WHO target, it is important to quantify current population salt intakes. This is particularly important in sub-Saharan Africa where the epidemiological transition is likely to result in dietary changes and a large increase in the prevalence of NCDs. In addition, resources for treatment of salt-associated diseases may not meet population needs so preventative strategies, such as salt reduction strategies, are key to averting the associated morbidity and mortality. To identify populations in which sodium intake is high, and to assess progress against the WHO sodium target, knowledge of sodium intake in sub-Saharan Africa is required.

Systematic reviews examining population salt intake globally have recently been conducted, however, the statistics reported for sub-Saharan African countries were limited [15, 16]. We re-examined this question focusing on sub-Saharan Africa only, in order to maximize the data gathered.

## Methods

### Search strategy

MEDLINE and Google Scholar were searched on March 2, 2015 using comprehensive search terms [Table 1]. No language limits were applied. While no date limits were applied in the MEDLINE search, the Google Scholar search was restricted to articles published from 1960 onwards. Reference lists of included studies were also searched to identify further studies.

**Table 1:** Search strategy

Medline:
1. Exp Sodium Chloride, Dietary/ or exp Sodium, Dietary/
2. Salt or sodium
3. Exp Africa/
4.1 OR 2
5. 3 AND 4
6. Limit to humans
Google Scholar:
Note: Words in brackets are combined with an “OR”
(Africa Angola Benin Botswana “Burkina Faso” Burundi Cameroon “Cape Verde” “Central African Republic” Chad Comoros Congo “Cote d’Ivoire” Djibouti “Equatorial Guinea” Eritrea Ethiopia Gabon Gambia Ghana Guinea Guinea-Bissau Kenya Lesotho Liberia Madagascar Malawi Mali Mauritania Mauritius Mozambique Namibia Niger Nigeria Reunion Rwanda “Sao Tome and Principe” Senegal Seychelles “Sierra Leone” Somalia “South Africa” Sudan Swaziland Tanzania Togo Uganda “Western Sahara” Zambia Zimbabwe) AND (salt sodium) AND (dietary diet intake urine urinary)

### Inclusion criteria

In order to be considered for inclusion, identified studies must have reported salt or sodium intake based on 24 h or timed urinary collection, or on dietary analysis. Studies reporting spot or overnight urine collection were not eligible for inclusion. Studies were required to include a general population sample or a normotensive population sample from sub-Saharan Africa.

### Data extraction and handling

Papers relating to the same study were examined together. Data was extracted independently by two researchers. This was done using a spreadsheet with the following headings:

1. Authors.
2. Year of publication.
3. Year of data collection.
4. Country of study.
5. Details of the sample.
6. Measurement used.
7. Quality assessment: Reliability and accuracy of measurement (after Powles and colleagues, 2013 [15]).
  - For urine collection (from high quality to low): *U1* – 24 h urine with PABA validation; *U2* – 24 h urine with exclusions based on observed/expected creatinine ratio or total urinary creatinine; *U3* – 24 h urine with other strict urine collection protocol without use of PABA or creatinine; *U4* – 24 h urine with other collection protocol or not recorded; *U5* – Less than 24 h urine, but timed in order to correct to 24 h
  - For dietary methods: *D1 –* Multiple short-term diet recalls; *D2* – Food Frequency Questionnaire; *D3* – Single dietary recall; *D4 –* Other (stated).
  8. Quality assessment – Representativeness of sample population: *A* – random sampling from explicitly stated sampling frame; *B –* other sampling strategy.
  9. Sample size.
  10. Sodium intake (g/day). We extracted data for adults and children, male and female, for urban and rural groups, and for black and non-black ethnicity separately, when these were reported. Where necessary we used the conversion of 1 g sodium = 43.5 mmol sodium, and 1 g sodium = 2.5 g salt. Where both dietary and urinary figures were available we extracted the urinary figures only.
  11. Standard deviation of the mean sodium intake.
  12. Standard error of the mean sodium intake.

Where there was any discrepancy arising in the data extraction it was resolved by the two reviewers (OO and SO). In cases in which data were not reported in published papers, corresponding authors were contacted to supply these results.

### Statistical analysis

Random-effects meta-regression was used to explore the role of sex, year of data collection, country’s economic development, location (urban or rural), and method of measurement (urinary or dietary) as sources of heterogeneity for the estimated salt intakes of adults. For these analyses we treated semi-urban and semi-nomadic populations as rural. Country was used to determine the level of economic development of the study setting based on World Bank definitions at the time of writing this paper [17].

Where year of data collection was not recorded, we assumed three years prior to publication, and where a period covering more than one year was recorded, we used the midpoint for the meta-regression analysis.

Where a standard error of the mean was not reported this was calculated using the standard deviation and number of study participants. In some studies either sample number or standard deviation were missing. If sample number was missing but could be approximated from data included in the paper, this was done. In cases where the range was reported, the standard error of the mean was estimated using the range rule [18].

Sensitivity analyses included excluding studies with the lowest quality measurement and excluding non-black populations (populations in sub-Saharan Africa but not of sub-Saharan African descent). The *metareg* package in Stata 13 was used for all analyses.

## Results

**Fig. 1:**
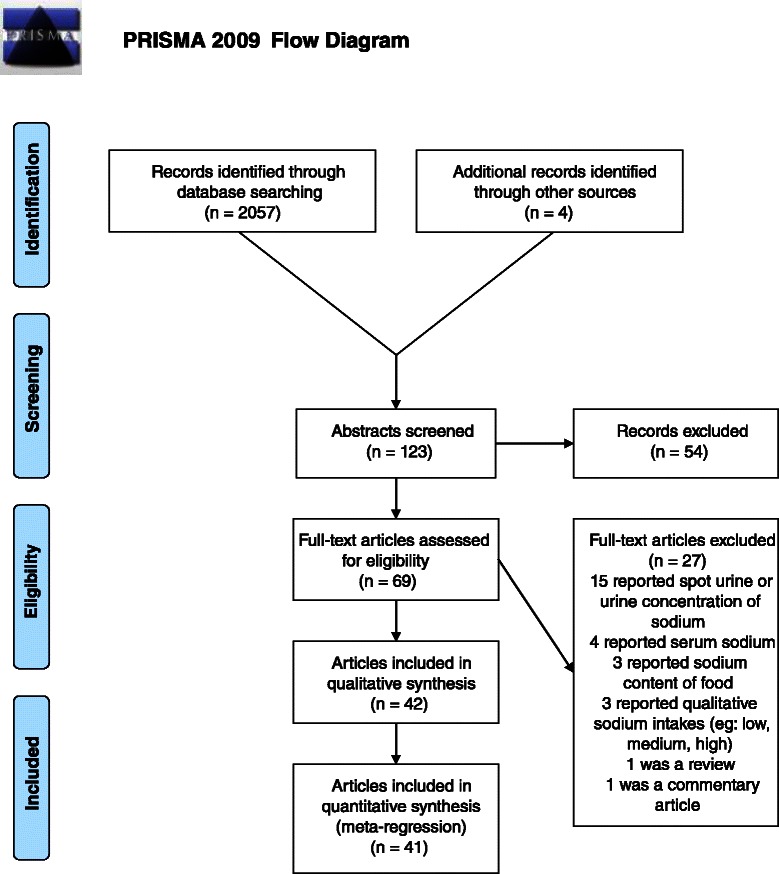
PRISMA 2009 flow diagram

Our search of MEDLINE found 1,057 titles. These were screened and 116 abstracts were retrieved after excluding irrelevant papers. After reading the abstracts, 54 papers were excluded, leaving 62 papers that were retrieved in full. Of these, 37 were found to be relevant. Searching Google Scholar returned 75,700 results. These were ordered by relevance and the first 1,000 titles were reviewed. Three additional papers were retrieved after reviewing these titles. Of these, two were excluded, leaving one paper that included additional reporting of a study previously identified in the MEDLINE search. An additional four papers were found through reference searches. This gave a total of 42 included papers reporting on 34 individual studies [19–60]. Figure 1 is a PRISMA flow chart giving details of the search. Table 2 gives details of included studies.

**Table 2:** Included studies

Country	Period of data collection	Population and Quality of Sample Representativeness	Method and Quality of Data Collection	References
Benin	January-February 1996	Male school children aged 6–12 and their mothers in rural Penessoulou, Atacora. (B).	U3	Melse-Boonstra et al, 1998 [19]
Botswana	October 1967, May 1968	Male adults recruited from the Kung bushmen in Northwestern Ngamiland. (B).	U4	Truswell et al, 1972 [20]
Cameroon	1993-1995	Random sample recruited from the civil service in Yaounde (affluent urban) and villages in a district in the same region (rural poor). Men and women aged 25–74. (A).	U4	Cooper et al, 1997 [21]
Cote d’Ivoire	Not reported	Families chosen by the investigators as representative of the populations recruited from 3 Northern villages (Koro, N’deo and Maranama) and from Abidjan. Children aged 2–12 and adults. (B).	D4 (all food eaten by the families for 3 consecutive days was weighed by investigators). U3 (adults only).	Hess et al, 1999 [22]
Ghana	February-April 2007	Random sample selected from a population register recruited from rural Kassena-Nankana District. (A).	D4 (household inventories)	Kunutsor and Powles, 2009 [23]
Ghana	June 2001-June 2002	Random sample selected from census, recruited from 12 villages, 6 semi-urban, 6 rural in the Ejisu-Juabeng and Kumasi Districts. Adults aged 40–75, 92 % Ashanti tribe, 94 % Twi-speaking. (A).	U4.	Cappuccio et al, 2006 [24]; Kerry et al, 2005 [25]
Ghana	June 1997-June 1999	Patients aged 5–12 in the paediatric surgery unit of the Korle-Bu Teaching Hospital, Accra, with surgical conditions that did not affect urine volume (herniae, hydroceles, undescended testes, hypospadias and tumours) and post-operative patients after surgery for acute appendicitis who had fully recovered. All eating a normal diet. Ready for discharge but detained for 24 h urine to be collected. (B).	U4	Badoe et al, 2005 [26]
Ghana	Not reported	Patients recruited from Korle-Bu Teaching Hospital, Accra, suffering from uncomplicated hernias, nodular goitre, breast tumours and simple tumours eating a full hospital diet. Adults aged 20–69, all ambulant, no evidence of urinary disease. (B).	U4	Badoe and Osafo, 1971 [27]
Kenya	1986	Random sample drawn from 320 households in the rural villages of Rambugu and Ndori, north of Lake Victoria in Western Kenya. Exclusively from the Luo tribe. Pregnant women excluded. (Part of INTERSALT). (A).	U3	Carvalho et al, 1989 [28]
Kenya	September 1980-November 1984	Recruited from two rural populations (the Luo tribe and the Kamba tribe) and migrants from the Luo community to Nairobi (urban migrants). Only spot urine done on Kamba population, therefore not included in this systematic review. (B).	U4	Poulter et al, 1985 [29]
Malawi	Not reported	All inhabitants from the first building estate in Lilongwe (urban, mainly white collar workers) and from the villages of Gunde and Msinje (rural, mainly farmers). Urine requested from men aged 15+ with even subject numbers. (B).	U4	Simmons et al, 1986 [30]
Nigeria	Not reported	Recruited from residential communities in South-western Nigeria. Excluding pregnant women and those on anti-hypertensives. (B).	U3	Tayo et al, 2012 [31]
Nigeria	November 2006-January 2007	All hypertensive patients attending the hypertensive clinic and selected normotensive controls attending the outpatient departments of two specialist hospitals in Akure and Ondo towns. Adults aged 20+. Normotensive controls only considered in this review. (B).	D3. U5.	Ijarotimi and Keshinro, 2008 [32]
Nigeria	Not reported	Recruited from two rural communities in Southwest Nigeria Igbo-Ora and Idere. Excluding pregnant or breastfeeding women and people with diabetes, kidney disease or atherosclerosis and BMI over 40. Normotensive men and women. Aged 25–55. (B).	U4	Forrester, 2005 [33]
Nigeria	Not reported	Free-living adult volunteers aged 18–48. University staff and/or their dependents of lower to high socio-economic status by Nigerian standards. (B).	D1	Smith, 1988 [34]
Nigeria	1993-1995	Random sample recruited from the rural village of Idere and 2 sites within Ibadan: Idikan traders and crafters (urban poor) and male pensioners of the Nigerian Railway Corporation (urban salaried). All Oyo Yoruba aged 25–74. (A).	U4	Kaufman et al, 1999 [35]; Cooper et al, 1997 [21]; Kaufman et al, 1996 [36]
Nigeria	Summer 1990	All civil servants in Sokoto, Northern Nigeria excluding pregnant women. Aged 18–66. (B).	U5	Bunker et al, 1996 [37]
Nigeria	Not reported	Random sample from two secondary day schools and one primary day school in Calabar (urban) and one co-educational secondary day school and one primary day school in Akpabuyo (rural). Boys and girls aged 12–14. (A).	U3	Ekpo et al, 1990 [38]
South Africa	Not reported	Random sample selected from housing map, recruited from rural communities of Empangeni, KwaZulu-Natal. Adults 19+ excluding pregnant or breast feeding women. (A).	D3	Kolahdooz et al, 2013 [39]
South Africa	2002-2006	Random sample selected from recent census, recruited from metropolitan areas of Johannesburg. Adults aged 17+ of black African descent. (A).	U2	Maseko et al, 2006 [40]; Millen et al, 2013 [41]; Redelinghuys et al, 2010 [42]
South Africa	2002	Convenience sample recruited from staff of the Cape Town City Council offices. Including hypertensive and normotensive adults aged 20–65 years old from three ethnic groups (black, white, mixed). Normotensive population only considered in this review. (B).	D1. U1.	Charlton et al, 2013 [43]; Charlton et al, 2008 [44]; Charlton et al, 2005 [45]; Charlton et al, 2005 [46]
South Africa	March 1981-October 1982	Adult patients with hypertension and normotensive controls recruited from two light industrial firms (urban Zulus and urban Indians), from the Lamontville township and the outpatient department of King Edward VIII hospital (urban Zulus), from a satellite clinic of KEVIII hospital (urban Indians) from Bethesda hospital in Ubombo, Benedictine hospital in Nongoma and others from the same communities (rural Zulus). Patients with complications of hypertension or major concomitant illness were excluded from the study. Normotensive controls only considered in this review. (B).	U5	Hoosen et al, 1985 [47]
South Africa	Not reported	Healthy male volunteers resident in Johannesburg. Aged 20–30 years. (B).	U2	Barlow et al, 1985 [48]
South Africa	1980	Random sample selected from male employees of a wire-rope manufacturing company near Johannesburg. Aged 30–50 years. (A).	U2	Barlow et al, 1982 [49]
South Africa	Not reported	Normotensive factory workers and hospital staff and hypertensive patients. Normotensive population only included in this analysis. (B).	U3	Cohen et al, 1982 [50]
South Africa	August-September 1978	Urban, apparently healthy, informed volunteers from domestic and clinical staff of the Groote Schuur Hospital, Cape Town and rural Xhosa volunteers from villages surrounding St Lucy’s Mission Hospital in the Transkei. Excluding those taking any drug (including oral contraceptives). (B).	D4 (“a full dietary history”)	Sever et al, 1980 [51]
Tanzania	Not reported	13–21 year old Bantu-speaking boys/young men from a secondary boarding school in Mafinga, Iringa District. (B).	D4 (analysis of weekly menu)	Rebacz-Maron et al, 2013 [52]
Tanzania	Not reported	20–50 year old healthy men living in Mwanza (urban). (B).	U2	Hamada et al, 2010 [53]
Tanzania	1998	Random sampling from administrative lists recruited from urban (Dar es Salaam), rural (Handeni) and semi-nomadic (Monduli) communities. Adults aged 47–57. (A).	U2	Njelekela, 2001 [54]
Tanzania	1987	Random sample recruited from Dar es Salaam (urban), Handeni (rural) and Moduli (nomadic-rural). Aged 30–54. (A).	U4	Mtabaji et al, 1990 [55]
Tanzania and Uganda	Not reported	General population cohorts from Lugarawa district in Tanzania and Lugbara in Uganda. (B).	D4 (detailed questionnaire about dietary habits)	Pavan et al, 1997 [56]
The Democratic Republic of the Congo	December 1983-May 1984	10 % random sample of a quarter of Kinshasa. Aged 10+. Subjects on anti-hypertensives were excluded. (A).	U2	M’Buyamba-Kabangu et al, 1986 [57]; M’Buyamba-Kabangy et al, 1986 [58]
Zimbabwe	Not reported	Male school children in rural Zimbabwe. (B).	U4	Matthews and Pegge, 1997 [59]
Zimbabwe	Not reported	Volunteer first year medical students. Male and female, average age 20 years. (B).	U2	Mufunda et al, 1992 [60]

The 34 studies were carried out in 13 countries and published between 1972 and 2013. Dates of data collection were not reported in all studies, but the earliest reported data collection was in 1967 and the most recent was in 2007. Twenty-seven studies examined adults only, four studies examined children only, and three included both adults and children.

**Fig. 2:**
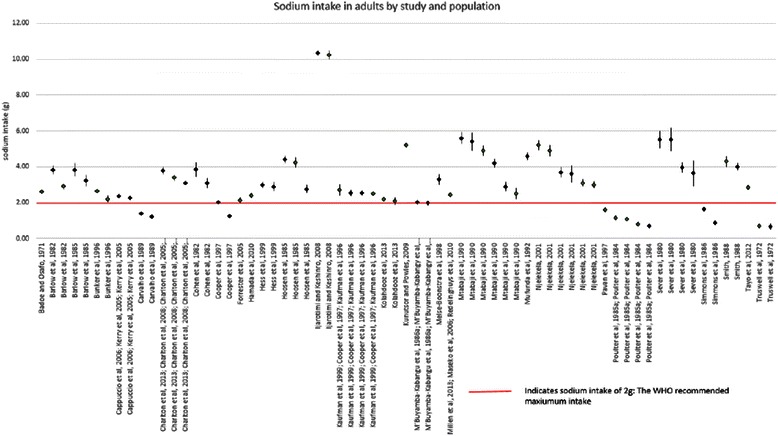
Sodium intake (g) in adults (see Table 3). Red line indicates sodium intake of 2 g, the WHO recommended maximum intake. Green markers indicate rural populations. Black markers indicate urban populations. Error bars show standard error of the mean

Table 3 and Fig. 2 shows sodium intake in adults reported from 30 studies. By extracting data for male and female, for urban and rural groups, and for black and non-black ethnicity separately (when these were reported), this gave 67 population estimates in total. Thirteen of 67 (19.4 %) populations studied had reported sodium intakes below the WHO recommendation. The two lowest sodium intakes were both found in rural Botswana in the 1960s. Of the others, six were found in Kenya in rural populations and recent migrants, and two from an urban and a rural population from Malawi, studied in the 1980s. The remaining three included one female urban population from the Democratic Republic of the Congo, studied in the 1980s; one urban population from the Cameroon; and one rural population from Tanzania and Uganda, studied in the 1990s.

**Table 3:** Sodium intake (g) in adults

		Men			Women			Both		
Study	Population	n	Mean	S.D.	n	Mean	S.D.	n	Mean	S.D.
Benin										
Melse-Boonstra et al, 1998 [19]	Rural	-	-	-	13	3.29	1.10	-	-	-
Botswana										
Truswell et al, 1972 [20]	Rural (1967)	6	0.71	N/S	-	-	-	-	-	-
Truswell et al, 1972 [20]	Rural (1968)	4	0.67	N/S	-	-	-	-	-	-
Cameroon										
Cooper et al, 1997 [21]	Rural	-	-	-	-	-	-	N/S	2.03	1.10
Cooper et al, 1997 [21]	Urban	-	-	-	-	-	-	N/S	1.25	0.69
Cote d’Ivoire										
Hess et al, 1999 [22]	Rural	-	-	-	-	-	-	51	2.90	1.90
Hess et al, 1999 [22]	Urban	-	-	-	-	-	-	52	3.00	1.30
Ghana										
Kunutsor and Powles, 2009 [23]	Rural	-	-	-	-	-	-	78	5.20	N/S
Cappuccio et al, 2006 [24]; Kerry et al, 2005 [25]	Rural	-	-	-	-	-	-	481	2.28	1.03
Cappuccio et al, 2006 [24]; Kerry et al, 2005 [25]	Semi-Urban	-	-	-	-	-	-	532	2.37	1.03
Badoe and Osafo, 1971 [27]	Urban	-	-	-	-	-	-	131	2.62	N/S
Kenya										
Carvalho et al, 1989 [28]	Rural	90	1.39	0.84	86	1.23	0.65	-	-	-
Poulter et al, 1985 [29]	Rural Luo	126	0.71	0.30	78	0.80	0.53	-	-	-
Poulter et al, 1985 [29]	Migrant Luo	78	1.15	0.55	61	1.08	0.51	-	-	-
Malawi										
Simmons et al, 1986 [30]	Urban	-	-	-	-	-	-	123	1.65	0.98
Simmons et al, 1986 [30]	Rural	-	-	-	-	-	-	78	0.86	0.71
Nigeria										
Tayo et al, 2012 [31]	-	-	-	-	-	-	-	804	2.85	1.26
Ijarotimi and Keshinro, 2008 [32]	-	203	10.23	3.42	249	10.34	2.37	-	-	-
Forrester et al, 2005 [33]	Rural	-	-	-	-	-	-	58	2.14	1.21
Kaufman et al, 1999 [35]; Cooper et al, 1997 [21]; Kaufman et al, 1996 [36]	Rural farmers (45+)	53	2.55	1.30	-	-	-	-	-	-
Kaufman et al, 1999 [35]; Cooper et al, 1997 [21]; Kaufman et al, 1996 [36]	Urban Poor (45+)	73	2.54	1.16	-	-	-	-	-	-
Kaufman et al, 1999 [35]; Cooper et al, 1997 [21]; Kaufman et al, 1996 [36]	Urban Salaried (45+)	18	2.72	1.34	-	-	-	-	-	-
Kaufman et al, 1999 [35]; Cooper et al, 1997 [21]; Kaufman et al, 1996 [36]	Combined (45+)	144	2.57	1.23	178	2.50	1.38	322	2.53	1.31
Kaufman et al, 1999 [35]; Cooper et al, 1997 [21]; Kaufman et al, 1996 [36]	Combined (25–74)	-	-	-	-	-	-	N/S	2.79	1.75
Bunker et al, 1996 [37]	Urban	378	2.63	1.82	59	2.21	1.50	-	-	-
Smith, 1988 [34]	Urban	7	4.01	0.49	9	4.30	0.89	16	4.18	0.53
South Africa										
Kolahdooz et al, 2013 [39]	Rural	51	2.08	1.41	84	2.20	0.80	-	-	-
Maseko et al, 2006 [40]; Millen et al, 2013 [41]; Redelinghuys et al, 2010 [42]	Urban	-	-	-	-	-	-	635	2.42	1.68
Charlton et al, 2013 [43]; Charlton et al, 2008 [44]; Charlton et al, 2005 [45]; Charlton et al, 2005 [46]	Urban White	-	-	-	-	-	-	103	3.79	2.09
Charlton et al, 2013 [43]; Charlton et al, 2008 [44]; Charlton et al, 2005 [45]; Charlton et al, 2005 [46]	Urban Mixed	-	-	-	-	-	-	112	3.39	1.69
Charlton et al, 2013 [43]; Charlton et al, 2008 [44]; Charlton et al, 2005 [45]; Charlton et al, 2005 [46]	Urban Black	-	-	-	-	-	-	110	3.11	1.15
Barlow et al, 1985 [48]	Urban White	11	3.83	1.21	-	-	-	-	-	-
Barlow et al, 1985 [48]	Urban Black	10	3.23	0.96	-	-	-	-	-	-
Hoosen et al, 1985 [47]	Urban Zulus	-	-	-	-	-	-	N/S	4.41	N/S
Hoosen et al, 1985 [47]	Rural Zulus	-	-	-	-	-	-	N/S	4.19	N/S
Hoosen et al, 1985 [47]	Urban Indians	-	-	-	-	-	-	N/S	2.76	N/S
Cohen et al, 1982 [50]	Urban White	-	-	-	-	-	-	17	3.84	1.70
Cohen et al, 1982 [50]	Urban Black	-	-	-	-	-	-	19	3.10	1.22
Barlow et al, 1982 [49]	Urban White	34	3.83	1.44	-	-	-	-	-	-
Barlow et al, 1982 [49]	Urban Black	71	2.91	1.27	-	-	-	-	-	-
Sever et al, 1980 [51]	Urban	N/S	5.52	1.31	N/S	5.52	1.72	15	5.52	1.52
Sever et al, 1980 [51]	Rural	N/S	3.95	0.74	N/S	3.63	1.84	15	3.72	1.59
Tanzania										
Hamada et al, 2010 [53]	Urban	74	2.38	1.17	-	-	-	-	-	-
Njelekela, 2001 [54]	Urban	81	5.20	24.80	79	4.90	2.70	-	-	-
Njelekela, 2001 [54]	Rural	93	3.10	1.80	91	3.00	1.80	-	-	-
Njelekela, 2001 [54]	Semi-Nomadic	41	3.60	3.00	61	3.70	2.30	-	-	-
Mtabaji et al, 1990 [55]	Urban	103	5.60	3.40	87	5.40	4.80	-	-	-
Mtabaji et al, 1990 [55]	Rural	88	4.90	2.70	96	4.20	2.40	-	-	-
Mtabaji et al, 1990 [55]	Semi-Nomadic	58	2.50	2.30	64	2.90	2.00	-	-	-
Tanzania and Uganda										
Pavan et al, 1997 [56]	Rural							370	1.6	-
The Democratic Republic of the Congo										
M’Buyamba-Kabangu et al, 1986 [57]; M’Buyamba-Kabangy et al, 1986 [58]	Urban	144	2.02	N/S	169	1.98	N/S	313	2.00	1.17
Zimbabwe										
Mufunda et al, 1992 [60]	Urban	-	-	-	-	-	-	55	4.60	1.53

The highest sodium intake was recorded in a Nigerian population in 2006 at over 10 g of sodium per day. It is likely, however, that these outliers are unreliable, as 24 h urine samples were not collected. Instead timed urines were used to calculate possible excretion of a 24 h time frame. Other high sodium intakes (over 5 g/day) were reported in populations in Tanzania, South Africa, and Ghana.

In studies that included estimates for both a rural and an urban population, the urban population estimates were higher in almost every case (Cameroonian estimates in Cooper and colleagues, 1997 [21] were one exception). In studies that included estimates for both male and female populations there were ten in which men consumed more salt than women, six in which women consumed more than men, and one in which their consumption was equal.

Two studies (Kunutsor and Powles, 2009 [23]; Pavan and colleagues, 1997 [56]) were excluded from the meta-regression as they did not report a standard error of mean or enough data to estimate it. Exploring five potential sources of heterogeneity separately suggested there was an association between year of data collection and salt intake; and between location (urban or rural) and salt intake at the 10 % level, with higher salt intakes in more recent studies and higher salt intakes in urban populations than in rural ones (Table 4).

**Table 4:** Meta-regression entering single covariates

Covariate	Coefficient	CI	p
Year of data collection	0.039	−0.003-0.081	0.071
Method of data collection (Urinary as reference)	0.801	−0.580-2.181	0.251
Sex (% men)	−0.103	−0.659-0.453	0.713
Economic development (1 = L, 2 = LM, 3 = UM)	0.212	−0.325-0.749	0.433
Location (Rural as reference)	0.790	0.142-1.438	0.018

Multivariate meta-regression showed a significant association between location and salt intake, with urban populations consuming higher levels of salt than rural populations; and between measurement used and salt intake, with dietary studies finding higher consumption of salt than urinary studies. These associations were robust in sensitivity analyses (Table 5).

**Table 5:** Meta-regression entering all covariates β coefficient (95 % confidence interval)

Covariate	Model 1	Model 2 (Excluding Q = U5)	Model 3 (Excluding non-black)
Year of data collection	0.012 (-0.020-0.044)	0.015 (-0.018-0.047)	0.012 (-0.023-0.048)
Method of data collection (Urinary as reference)	1.136 (0.066-2.206) *	1.288 (0.211-2.365)*	1.275 (0.126-2.422)*
Sex (% men)	−0.061 (-0.467-0.345)	−0.044 (-0.466-0.379)	−0.064 (-0.497-0.368)
Economic development (1 = L, 2 = LM, 3 = UM)	−0.034 (-0.446-0.378)	−0.159 (-0.599-0.281)	−0.124 (-0.611-0.362)
Location (Rural as reference)	0.874 (0.215-1.534)*	1.010 (0.326-1.694)*	0.814 (0.105-1.523)*

*significant at *p* < 0.05

Table 6 shows sodium intake in children. Urban child populations in Ghana, Nigeria, and the Democratic Republic of Congo were consuming more than the adult recommended intake of salt, as was a population of rural children in Benin. The highest recorded sodium intake was in Nigerian girls aged 12–14 who consumed 3.85 g per day. The lowest sodium intake was found in a male rural population from Zimbabwe who consumed just 0.92 g per day. In each of the three populations for which girls’ and boys’ sodium intake was reported separately, girls had a higher sodium intake than boys.

**Table 6:** Sodium intake (g) in children Mean (S.D.) *n*

	Urban			Rural		
Study	Boys	Girls	Both	Boys	Girls	Both
Badoe and colleagues, 2005 [26]	-	-	2.50 (-) *74*	-	-	-
Ekpo and colleagues, 1990 [38]	1.87 (0.94) *20*	3.85 (1.79) *20*	-	1.59 (0.75) *19*	1.79 (0.74) *19*	-
Hess and colleagues, 1999 [22]	-	-	-	-	-	1.78 (0.72) *84*
Matthews and Pegge, 1997 [59]	-	-	-	0.92 (0.65) *32*	-	-
M’Buyuma-Kabangu, 1986 [57]	1.79 (-) *87*	2.02 (-) *113*	1.93 (-) *200*			
Melse-Boonstra and colleagues, 1998 [19]	-	-	-	2.71 (0.99) *13*	-	-
Rebacz-Maron and colleagues, 2013 [52]	1.25 (-) *91*	-	-	-	-	-

## Conclusions

Through extensive systematic searching focusing on sub-Saharan Africa and with no date restrictions, we found 42 papers reporting sodium intakes in sub-Saharan African populations, including seven that examined children. The previous systematic reviews in global populations mentioned in the introduction (Brown and colleagues, 2009 [16]; and Powles and colleagues, 2013 [15]) examined papers published between 1988-2008 and 1980-2011, and identified five and 11 papers reporting sodium intakes in sub-Saharan African populations, respectively. Brown and colleagues [16] did not find any estimates for African children, while Powles and colleagues [15] did not include children in their systematic review.

We have found that sodium intake in many adult populations in sub-Saharan Africa is above the 2 g intake recommended as an upper limit by the WHO, and also above this limit in some populations of children. Indeed, there have been no estimates of sodium intake for adult populations that fell below this 2 g limit reported since the 1990s. Through meta-regression analyses and by doing a within-study comparison, we have found that sodium intake is likely to be higher in urban than in rural populations. This is an important finding because of the trend for urbanization in sub-Saharan Africa, which will put increasing numbers at risk of hypertension and its sequelae, if this association is robust.

Meta-regression also suggested that dietary methods for assessing sodium intake might give higher estimates of sodium consumption than urinary methods in sub-Saharan African settings. Estimates of sodium intake based on dietary and urinary methods of estimation each have specific strengths and weaknesses. Although 24 h urinary excretion is not prone to reporting biases, participant burden is high and this may lead to attrition bias (if quality standards for acceptable collection are stringent) or measurement bias due to incomplete or over-collected urine (where quality standards are lower). In addition, 24 h urinary excretion takes no account of loss of sodium through other means, for example through feces and/or sweat. Timed urine collection allows for a lesser participant burden, but may be biased because of diurnal variation in sodium excretion [61]. Dietary estimates of sodium intake might not be accurate due to recall bias, reporting errors, erroneous food composition tables (for example, because they are not country specific or because they are out-of-date), and/or difficulty in quantifying added salt (including, for example, salt added during cooking but discarded in cooking water, rather than consumed). Our finding that sodium intake estimates are higher based on dietary rather than urinary measures is the opposite of that reported elsewhere [62]. Others have found that estimates based on food diaries, weighed records, food-frequency questionnaires, and 24 h dietary recall underestimated sodium intakes compared with 24 h urine collections. It is worth noting though, that despite the meta-regression results, in each of the three included papers that used both dietary and urinary estimates of sodium intake [22, 32, 46], the estimate from dietary data was lower than from urinary data. It is conceivable that in sub-Saharan Africa more sodium is lost through routes other than urinary excretion, for example, through sweat. This is worth considering given that Powles and colleagues [15] found that African sodium intakes (although above WHO recommendations) were lower than the rest of the world, based on 24 h urinary excretions, uncorrected for non-renal losses.

We did not find a difference between the sexes in sodium intake – in contrast to both global systematic reviews [15, 16] that found men consumed more than women, and boys consumed more than girls. In the child populations identified in our review, girls consumed more sodium than boys, though this finding is based on just two studies with fairly small samples sizes. There is no apparent reason why there would be a different association with sex in sub-Saharan Africa than in the rest of the world. However, the fact that there may be a difference suggests that context-specific research needs to be done to establish whether patterns of sodium intake in sub-Saharan African settings are similar to or different from high-income countries or other LMICs.

In this study we have not examined potassium intake, which may mitigate the effects of high sodium on blood pressure. Understanding sodium intake in sub-Saharan Africa in the context of the whole diet could give additional levers with which to tackle cardiovascular disease risk.

Of 66 estimates of sodium intake for adults identified in this review, only 20 were given a measurement quality score of 1 or 2, and just 28 were rated grade A for sampling strategy. This demonstrates that high quality studies of sodium intake in Africa remain rare. It also means that the implications of our results are limited – it is possible that sodium intake in sub-Saharan African countries differs to the estimates reported here. High quality measurements in representative samples of the general population will be required to identify where and with whom to intervene in order to meet the WHO target of a 30 % reduction in population sodium intake and to demonstrate progress towards this target.

## Abbreviations

CVD: cardiovascular disease
LMICs: low- and middle-income countries
NCD: non-communicable disease
WHO: World Health Organization
